# Effectiveness of the SMART training intervention on ankle joint function in patients with first-time acute lateral ankle sprain: study protocol for a randomized controlled trial

**DOI:** 10.1186/s13063-023-07195-2

**Published:** 2023-03-03

**Authors:** Janina Tennler, Christian Raeder, Arthur Praetorius, Tobias Ohmann, Christian Schoepp

**Affiliations:** 1 Department for Arthroscopic Surgery, Sports Traumatology & Sports Medicine, BG Klinikum Duisburg, Duisburg, Germany; 2 Research Department, BG Klinikum Duisburg, Duisburg, Germany; 3 Department of Trauma and Reconstructive Surgery, University Hospital, Essen, Germany

**Keywords:** Lateral ankle sprain, Chronic ankle instability, Diagnostic, Prevention, Therapy

## Abstract

**Background:**

The lateral ankle sprain (LAS) is the most common injury in the field of everyday and sports-related activities. There is a high rate of patients with LAS who will develop chronic ankle instability (CAI). A possible explanation for this high rate is an insufficient rehabilitation and/or a premature return to intense exercise and workloads. Currently, there are general rehabilitation guidelines for LAS but there is a lack of standardized evidenced-based rehabilitation concepts for LAS, which effectively reduce the high CAI rate. The primary aim of the study is to investigate the effectiveness of a 6-week sensorimotor training intervention (SMART-Treatment, SMART) in contrast to standard therapy (Normal Treatment, NORMT) after an acute LAS on perceived ankle joint function.

**Methods:**

This study will be a prospective, single-center, interventional randomized controlled trial with an active control group. Patients (14–41 years) with an acute LAS and a MRI confirmed lesion or rupture of at least one ankle ligament will be included. Exclusion criteria are acute concomitant injuries of the ankle, pre-injuries of the ankle, serious lower-extremity injuries of the last 6 months, lower-extremity surgery, and neurological diseases. The primary outcome measure will be the Cumberland Ankle Instability Tool (CAIT). Secondary outcomes include the Foot and Ankle Ability Measurement (FAAM), isokinetic and isometric strength diagnostics, joint repositioning sense, range of motion, measurements of postural control, gait and run analysis, and jump analysis. This protocol will follow the SPIRIT guidance.

**Discussion:**

Current management of LAS rehabilitation lacks since there is a high rate of patients developing a CAI. It has been shown that exercise therapy improves ankle function in acute LAS as well as in patients with CAI. It is further recommended to address specific impairment domains in ankle rehabilitation. However, empirical data for such a holistic treatment algorithm is missing. Therefore, this study has the potential to improve the healthcare for LAS patients and might be used for a future standardized evidence-based rehabilitation concept.

**Trial registration:**

“Prospectively registered” ISRCTN – ISRCTN13640422 17/11/2021; DRKS (German Clinical Trials Register) – DRKS00026049.

**Supplementary Information:**

The online version contains supplementary material available at 10.1186/s13063-023-07195-2.

## Administrative information

Note: the numbers in squared brackets in this protocol refer to SPIRIT checklist item numbers. The order of the items has been modified to group similar items (see http://www.equator-network.org/reporting-guidelines/spirit-2013-statement-defining-standard-protocol-items-for-clinical-trials/).

**Table:** 

Title [1]	Effectiveness of the SMART training intervention on ankle joint function in patients with first-time acute lateral ankle sprain: study protocol for a randomized controlled trial
Trial registration [2a and 2b].	‘prospectively registered’ ISRCTN – ISRCTN13640422 17/11/2021 https://www.isrctn.com/ISRCTN13640422 DRKS (German Clinical Trials Register) – DRKS00026049 https://drks.de/search/en/trial/DRKS00026049
Protocol version [3]	Version 1 (01.01.2022)
Funding [4]	DGUV (German Social Accident Insurance) FF-FR 0329
Author details [5a]	Janina Tennler^1^, Christian Raeder^1^, Arthur Praetorius^1^, Tobias Ohmann^2^ and Christian Schoepp^1^ ^1^ Department for Arthroscopic Surgery, Sports Traumatology & Sports Medicine, BG Klinikum Duisburg, Germany ^2^ Research Department, BG Klinikum Duisburg, Germany **Authors ‘ contributions** JT:Ethics proposal, Protocol development, Writing – Original Draft, Writing – Review & Editing CR:Study design, Ethics proposal, Protocol development, Supervision, Writing – Review & Editing AP:Supervision, Writing – Review & Editing TO:Project administration, Writing – Review & Editing CS:Trial sponsor, Writing – Review & Editing
Name and contact information for the trial sponsor [5b]	Dr. Christian Schoepp BG Klinikum Duisburg, Klinik für Arthroskopische Chirurgie, Sporttraumatologie und Sportmedizin (Departement for Arthroscopic Surgery, Sports Traumatology & Sports Medicine) Großenbaumer Allee 250 47,249 Duisburg, Germany + 49 (0)203 7688 2745 Christian.schoepp@bg-klinikum-duisburg.de
Role of sponsor [5c]	This funding source had no role in the design of this study and will not have any role during its execution, analyses, interpretation of the data, or decision to submit results.

## Introduction

### Background and rationale [6a]

The lateral ankle sprain (LAS) is the most common injury in the field of everyday and sports-related activities [1]. LAS account for 15% of all reported injuries in NCAA (National Collegiate Athletic Association) sports [2]. Court sports like basketball, handball, and volleyball have the highest incidence rates with 7 ankle sprains per 1000 exposures [3]. According to Kemler et al., there is an increase in population-based data of medical and non-medical treated ankle injuries from 8.2 (2000) to 17.5 (2010) per 1000 person-years [4]. Ankle injuries are not limited to any specific group but affect the general population.

Ankle injuries can have severe consequences for the injured individual in terms of treatment costs and time-loss in work and sporting activities [1, 5]. The total costs of a LAS in the European Union range between 800 € and 1000 € [1]. LAS does not only affect individual health condition but also individual socioeconomic environment. In the Netherlands, absence from work was responsible for up to 80% of the total costs of a LAS [6]. Most of the cost analyses, however, just focus on the short-term treatment and management of LAS, although they are often just the beginning of persistent medical problems.

According to a systematic review, 25% of patients with LAS will develop a chronic ankle instability (CAI) [7]. This condition is characterized by a history of one significant LAS, subsequent recurrent sprains, episodes of the ankle giving way, or self-reported deficits in ankle function [8]. It is well established that the pathology of CAI is complex and associated with many factors such as mechanical insufficiencies like ligament laxity, arthrokinematic restrictions and/or functional insufficiencies like neuromuscular inhibition, altered reflexes, or diminished somatosensation [9]. Besides symptoms of subjective instability, recurrent sprains, and persistent pain, there are a range of impairments that were found in patients with CAI [7, 9, 10]. These impairments lay within range of motion (ROM), postural control, strength, and altered movement patterns and are described as followed.

Having a physiological ROM is substantial for performing functional tasks like walking or running without altering lower leg kinematics. The literature shows that both active and passive dorsiflexion ROM is limited following an acute LAS [11]. A pattern of decreased dorsiflexion ROM and restricted posterior talar glide is also observed in patients with CAI [11]. These limitations in arthrokinematic motion are highly recommended to be treated [12].

The relationship between impairments of postural control and CAI has been widely proven [9, 13, 14]. The most common assessments are the single-leg stance and the Star Excursion Balance Test (SEBT) or the modified Star Excursion Balance Test (Y-Balance or mod. SEBT). Significant differences in center of pressure (COP) analyses in the single-leg stance with eyes open and closed conditions were found between groups of CAI and healthy controls [13, 14]. This can be attributed to the decreased use of somatosensory input in patients with CAI. To compensate for it, there is a shift to a higher dependence on visual input to maintain postural control and therefore joint stability [15, 16].

Meta-analyses have identified concentric eversion, inversion, and plantarflexion strength deficits as well as eccentric eversion, inversion, plantarflexion, and dorsiflexion strength as contributor to CAI [17–19]. Muscle weakness in patients with CAI, however, is not limited to just the ankle muscles but also to the more proximal joints. There are deficits in concentric knee flexion and extension, isometric hip abduction and extension, external rotation, and eccentric hip flexion [10]. However, variability exists with measurement devices and methodology.

Differences between patients with CAI and healthy controls can further be seen in altered movement patterns as well as altered neuromuscular activation strategies during functional activities like walking or jumping. A recent review identified greater inversion and plantarflexion as well as a more laterally deviated center of pressure throughout the stance phase in walking [20]. During jumps, patients with CAI landed with a more dorsiflexion angle and therefore had less sagittal plane motion during the absorption phase of landing. This resulted in higher peak vertical ground reaction forces and faster loading rates due to the stiffer landing strategy [21]. During drop-vertical-jumps, Herb et al. found altered kinematic landing strategies which as well were less sagittal plane motion in the contact phase and further greater inversion angles [22]. On a neuromuscular level, Feger et al. identified altered activation strategies in patients with CAI during walking, representing an earlier onset of muscle activity including the tibialis anterior, peroneus longus, rectus femoris, biceps femoris, and gluteus medius [23]. Further, there was higher peroneus longus muscle activation before and during landing [22, 24]. Delahunt et al. [25] already suggested that individuals with CAI develop an altered, protective “feed-forward” motor-control pattern in preparation for initial contact. Since patients with CAI contact the ground in more inverted positions, the sensory experience of this “higher-risk-position” could be used to preprogram protective muscle activity and therefore prevent an ankle sprain, which is evidenced by an increase in peroneus longus muscle activation [25].

A possible explanation for the high rate of CAI is an insufficient rehabilitation and/or a too early return to intense sports and workloads after LAS [1, 26]. The ankle sprain is further often classified as a benign injury with the expectation to heal within a couple days. This results in an inappropriate early return to sports and stress on tissues [27]. Currently, there are general guidelines for the rehabilitation after LAS but there is no standardized evidenced-based rehabilitation concept for LAS, which effectively reduces the high CAI rate. Doherty et al. concluded that exercise therapy improves self-reported function following LAS and is generally considered effective in the treatment of CAI, especially if it is given in high doses (> 900 min of exercise therapy) [28]. Systematic reviews found that proprioceptive exercises as well as neuromuscular interventions (as part of exercise therapy) were able to reduce subjective instability and functional outcomes [29, 30]. Van Rijn et al. concluded that the addition of supervised exercise focusing on strength, mobility, and balance exercise compared to conventional treatment leads to a faster and better recovery after acute LAS [31]. In general, it is recommended to implement exercises that restore the normal range of motion (ROM), strength [32], and postural control [28, 32, 33]. However, empirical data for such a holistic treatment algorithm is missing.

For the treatment of patients with CAI, a systematic review from de Vries et al. concluded that neuromuscular training compared to no training results in better functional outcomes in patients with CAI [34]. However, according to a review from O’Driscoll et al., there was just moderate or limited evidence for the effectiveness of neuromuscular training (proprioceptive and strength training drills) on sensorimotor and functional deficits in patients with CAI [35]. Regarding self-reported function and reinjury incidence in CAI, exercise therapy is generally considered effective [28]. However, much of the literature exploring treatment interventions has examined the efficiency of a single treatment strategy instead of a comprehensive rehabilitation approach [36–38]. Hale et al. evaluated the efficacy of a 4-week comprehensive rehabilitation program for patients with CAI addressing ROM, muscle performance, and neuromuscular control. They were able to reduce lower extremity reach deficits as well as perceived deficits in activities of daily life and sport-specific skills [39].

As stated earlier, there is no evidence-based rehabilitation concept for acute LAS being able to reduce the long-term CAI rate. Such a program would not only improve the patients’ health status but also reduce socioeconomic costs [8]. Miklovic et al. were able to identify impairments that were both present in patients with CAI and patients with acute LAS. These impairment domains (ROM, postural control, strength, altered movement strategies) should be addressed in LAS rehabilitation [11]. Currently, this has not been proven yet.

The prevention of CAI is not only based on an evidenced-based rehabilitation concept but also on the early diagnostic of risk factors. According to the CAI model of Hertel, it is suggested to identify clinically relevant functional insufficiencies on individuals, since they might differ [40]. Delahunt and Remus concluded that being unable to perform a drop landing and a drop vertical jump within 2 weeks after initial injury as well as poorer dynamic postural balance and lower level of self-reported function 6 months post-injury can be associated with the development of CAI [5]. Yet there is no effective test battery, which can be used to aid diagnosis of functional ankle instability. Identifying functional ankle instability or predictors of CAI as early as possible is an important goal to prevent the development of high CAI rates [11]. Currently, there is a lack of evidence for a combined testing and training approach in the systematic treatment of acute LAS that effectively reduces the CAI rate.

### Objectives [7]

The *primary aim* of the study is to investigate the effectiveness of a sensorimotor training intervention (SMART-Treatment, SMART) in contrast to standard therapy (Normal Treatment, NORMT) after an acute first-time LAS on perceived ankle joint function. We hypothesize that patients treated with SMART show a better subjective outcome in perceived ankle joint function than patients treated with NORMT.

The *secondary aim* is to determine an effective test battery for the valid measurement of a functional ankle instability. This will be achieved by comparing injured patients (2 weeks after initial injury, t1, as well as after the training intervention, t2) with an uninjured control group (CONTROL).

While primary aim of the study is studied in a longitudinal manner, the design of the secondary aim of the study can rather be classified as a cross-sectional study. Therefore, both study arms work independent from each other, and each have an own statistical approach.

### Trial design [8]

This study will be a prospective, single-center, interventional randomized controlled trial with an active control group and a superiority framework. Furthermore, it will be unblended with the patient allocated in a 1:1 ratio to one of the two parallel groups.

## Methods: participants, interventions, and outcomes

### Study setting [9]

The study is carried out at the BG Klinikum Duisburg which is a supraregional trauma center belonging to the BG Kliniken Hospital Group (“BG = Berufsgenossenschaftliche”) of the German Federal Statutory Accident Insurance (“Deutsche Gesetzliche Unfallversicherung”) in Germany in the clinic for arthroscopic surgery, sports traumatology, and sports medicine.

### Eligibility criteria [10]

#### Inclusion criteria

Patents must meet the following criteria to be eligible for the study:

- ICD-Code S93.4x “sprain of the ankle”
- 14–41 years
- BMI 19–30
- MRI confirmed lesion or rupture of at least one lateral ankle ligament

#### Exclusion criteria

If the patients meet any of the following criteria, they will not be eligible for the study:

- Acute concomitant injuries of the ankle (fractures, syndesmosis ligament injury, osteochondral lesions)
- Pre-injuries of the injured and non-injured ankle
- Serious lower-extremity injuries of the last 6 months (e.g., fractures, ligament ruptures)
- Lower-extremity surgery (e.g., ACL-reconstruction)
- Neurological diseases or impairments of the vestibular system which could influence the physiological performance

### Who will take informed consent? [26a]

All patients admitted to the hospital with an acute ankle sprain are screened for participation. After the patient has been assessed as eligible by the responsible physicians and sport scientists, written informed consent to participate in the trial will be obtained. The documents will be stored according to the hospital’s commitment to GCP and GSP standards and the proceedings outlined in the trial’s ethical approval (Ärztekammer Nordrhein ethics commission, ref: 2021236). The informed consent contains information about the aim of the study and its timeline. There is a detailed description of all intervention groups and the content of the diagnostic. The participants are further informed about their financial compensation, potential risks, and their right to leave the study at any time and for any reason if they want. Additional information about casualty insurance as well as data protection are part of the informed consent. For potential participants under 18, their legal guardian has to sign the informed consent.

### Additional consent provisions for collection and use of participant data and biological specimens [26b]

Not applicable. There are no plans for ancillary studies that may use the data generated in this trial.

## Interventions

### Explanation for the choice of comparators [6b]

The comparator (Normal Treatment, NORMT) is the current standard therapy after having an acute LAS. In this group, the participants receive physiotherapy as a standard therapy, if necessary, to reduce swelling and pain as well as to improve ankle mobility. However, this therapy does not seem to sufficiently reduce the high rates of CAI [12, 41].

### Intervention description [11a]

The SMART intervention was developed to provide a systematic rehabilitation concept for acute LAS. It is a sensorimotor training program which addresses various functional training domains to regain ankle joint function and stability [5, 11]. SMART is an acronym and consists of the following domains: S = Sensory Stimulation, M = Mobilization, A = Activation and Balance, R = Resistance and Re-Integration, T = Transfer to Function and Performance. A typical rehabilitation process runs through different phases with different goals. Therefore, there is a progress in focus between the five domains over the 6 weeks, as described below: The domains S and M are present across the whole intervention. In week 1 and 2, the main focus is on the A domain, in weeks 3 and 4 on the R domain, and in weeks 5 and 6 on the T domain.

During the 6-week intervention period, five training sessions are held per week each lasting approximately 30–45 min. The detailed training plan of the SMART intervention can be found in the supplemental material. In the beginning, the training sessions will be center-based, to ensure individual feedback and to give participants the possibility to familiarize with the training concept. Across the 6 weeks, the training is shifting towards more home-based sessions, since the participants do not need the close supervision anymore (weeks 1–2: 6 × center-based and 4 × home-based; weeks 3–4: 4 × center-based and 6 × home-based; weeks 5–6: 2 × center-based and 8 × home-based). There are regularly video calls with the sport scientist to meet up with the participants and provide feedback.

### Criteria for discontinuing or modifying allocated interventions [11b]

Patients can leave the study at any time and for any reason if they want. In case of potential problems during the diagnostic, e.g., not being able to perform a jump, the tests will be adapted or skipped. This will be documented as “not feasible.”

### Strategies to improve adherence to interventions [11c]

#### Adherence to SMART

Participants receive detailed training information and additional training equipment for the SMART intervention to improve adherence. This consists of a training booklet with all exercise modalities as well as individualized video playlists for all exercises. Additionally, the training will be monitored by a sport scientist and verbal encouragement is given throughout the center-based and video call training sessions, respectively. Second, the participants can document their training load in the training booklet. As introduced earlier, the training will shift from center-based to home-based over the 6 weeks. This is to make sure that the participants can integrate in training into their everyday life and do not have to get to the study center for each training session. Third, the participants will get financial compensation after 6 weeks (50 €) and additionally 12 months (50 €).

#### Adherence for NORMT

To improve adherence, the participants will get a financial compensation after 6 weeks (25 €) and 12 months (25 €), respectively.

### Relevant concomitant care permitted or prohibited during the trial [11d]

Participants are informed that additional training or taking on participation in sports are forbidden during the trial.

### Provisions for post-trial care [30]

A casualty insurance will be granted to all participants of the study. However, there will be no provisions for post-trial care.

### Outcomes [12]

#### Primary outcome measurement

The primary outcome measurement is the subjectively perceived ankle joint function. This is measured by the standardized questionnaire Cumberland Ankle Instability Tool (CAIT). The difference in change in score between two groups will be assessed. The method of aggregation is mean ± SD, and the CAIT will be measured before and after the 6-week intervention as well as in the 6-month and a 12-month follow-up (Table 1).

**Table 1:** Primary and secondary outcome measurements

Primary	Secondary
CAIT	FAAM
	mod. SEBT and COP
	Joint repositioning sense
	ROM
	Gait and run analysis
	Jump analysis
	Strength diagnostic

#### Secondary outcome measurement

Secondary outcome measurements include the subjective Foot and Ankle Ability Measure (FAAM) by means of a psychometric questionnaire, isokinetic and isometric strength diagnostics, joint repositioning sense, ROM, measurements of postural control (mod. SEBT and COP), gait and run analysis, and jump analysis (Table 1).

The physical function for individuals with foot- and ankle-related impairments was measured by the standardized FAAM questionnaire. The difference in change in score between two groups will be assessed. The method of aggregation is mean ± SD, and the FAAM will be measured before and after the 6-week intervention as well as in the 6-month and a 12-month follow-up.

The *strength diagnostic* is assessed with a motorized dynamometer and can be divided into two parts: closed kinetic chain leg press measurements (N) and open kinetic chain plantarflexion/dorsiflexion measurements (Nm). The difference in change in strength between two groups will be assessed. The method of aggregation is mean ± SD and the strength diagnostics will be performed before and after the 6-week intervention as well as in the 12-month follow-up.

*Joint repositioning sense* of the plantarflexion/dorsiflexion movement is determined by the participant’s ability to actively replicate a passively placed joint reference angle. The difference in change between two groups will be assessed. The method of aggregation is mean ± SD, and the diagnostics will be performed before and after the 6-week intervention as well as in the 12-month follow-up.

*ROM* measurements will be performed in a isokinetic device. It is determined by the maximal passive and active plantarflexion and dorsiflexion. The difference in change in ROM between two groups will be assessed. The method of aggregation is mean ± SD, and the diagnostics will be performed before and after the 6-week intervention as well as in the 12-month follow-up.

*Dynamic postural control* will be assessed during the mod. SEBT. Measurements (cm) are taken in the anterior, posterior-medial, and posterior-lateral reach direction and are normalized to leg length resulting in a composite score. The difference in change in dynamic postural control will be assessed. The method of aggregation is mean ± SD, and the diagnostics will be performed before and after the 6-week intervention as well as in the 12-month follow-up.

*Static postural control* will be assessed during single-leg stance. Trials are performed with eyes open and closed with each leg (10 s each). For analysis, the projected COP is calculated and tracked during each trial. We will track the COP area (mm^2^), mean COP velocity (mm/s), and the COP excursion length (mm). Additionally, there will be time-to-boundary (TTB) analysis during the assessment of static postural control. The difference in change in static postural control between two groups will be assessed. The method of aggregation is mean ± SD, and the diagnostics will be performed before and after the 6-week intervention as well as in the 12-month follow-up.

There are two types of *jumps* that are performed: a unilateral drop landing and the bilateral drop jump. For both jumps, basic performance parameters (jump height, contact time, and the resulting reactive strength index (RSI)) are calculated. The difference in change between two groups will be assessed. The method of aggregation is mean ± SD and the diagnostics will be performed before and after the 6-week intervention as well as in the 12-month follow-up.

*Kinematic* analysis includes joint angles (°) for the sagittal, frontal, and transverse plane during jump, gait, and running analysis. The difference in change between two groups will be assessed and computed with statistical parametric mapping (SPM). The method of aggregation is mean ± SD, and the diagnostics will be performed before and after the 6-week intervention as well as in the 12-month follow-up.

*Kinetic* analysis includes force (N) development during jump, gait, and running analysis. The difference in change between two groups will be assessed and computed with SPM. The method of aggregation is mean ± SD, and the diagnostics will be performed before and after the 6-week intervention as well as in the 12-month follow-up.

*Electromyographic* analysis includes recordings of the tibialis anterior, peroneus longus, gastrocnemius medialis, and rectus femoris during jump, gait, and running analysis. The difference in change between two groups will be assessed and computed with SPM. The method of aggregation is mean ± SD, and the diagnostics will be performed before and after the 6-week intervention as well as in the 12-month follow-up.

### Participant timeline [13]

**Fig. 1:**
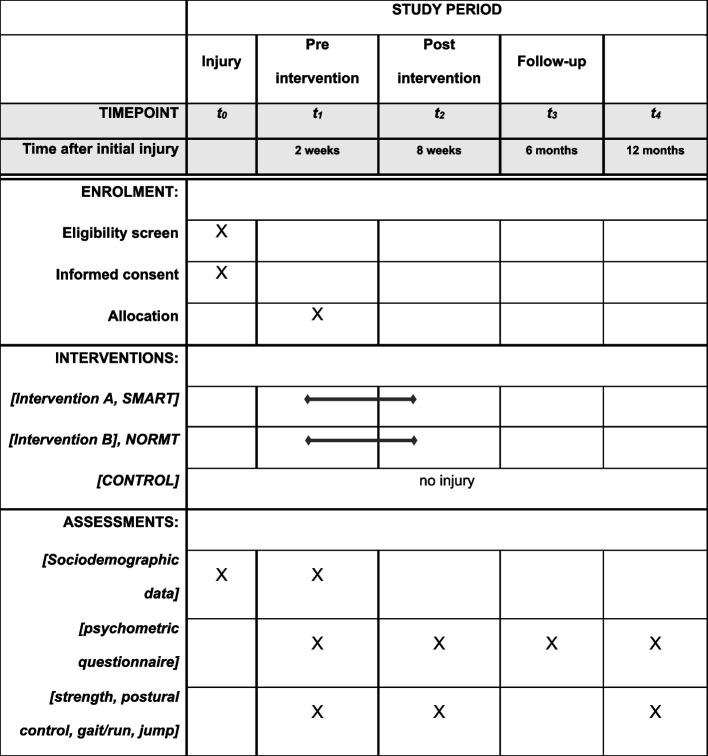
Schedule of enrolment, interventions, and assessments

The process of enrolment (eligibility screen, informed consent, basic sociodemographic data) will take place in the first 2 weeks after initial injury. At t1, before the 6-week intervention, participants will be allocated and there will be psychometric questionnaires as well as all strength, postural control, gait/run, and jump testing. The same will be done at t2 after the intervention. While t3 is just a follow-up done by email, t4 includes the full diagnostic standard as in t1 and t2 (Fig. 1).

### Sample size [14]

The sample size was calculated for the primary outcome (CAIT) and a repeated-measurement ANOVA with G*Power [42]. Therefore, the following assumptions were used: effect size *f* = 0.5, *α* = 0.05, statistical power (1 − *ß* = 0.80), two groups (SMART and NORMT), and four measurements (t1, t2. t3, t4). The effect size was set according to Wright et al. [43]. This resulted in a total sample size of 82 patients (2 × *n* = 41). With an accounted lost to follow-up of 20%, a sample size of 102 (2 × *n* = 51) is necessary to prove statistic group differences.

### Recruitment [15]

Participants will mainly be recruited through the emergency department and a cooperating outpatient medical center. All patients with acute LAS (ICD-Code S93.4x) are screened as a potential participant of the study. Patients without ankle fractures (investigated through X-ray) are filtered by further inclusion criteria and contacted by the sport scientists. The patients are specifically asked for their current symptoms. Only if two out of the three criteria (pressure pain on the lateral ankle/ swelling on the lateral ankle/ pain during activities of daily life) are met, additional MRI diagnostics is warranted (not part of standard diagnostic for ankle sprains in Germany). Confirmation of LAS by MRI scans finally leads to patient randomization and inclusion into the study.

## Assignment of interventions: allocation

### Sequence generation [16a]

We created a randomization plan using a computer-generated randomization schedule (https://www.graphpad.com/quickcalcs/randomize1/) to create a simple balanced randomization. Participants will be randomly assigned to either control or experimental group with a 1:1 allocation (2 × 41 for SMART and NORMT). Subsequent allocation is provided in a separate document that is unavailable to the sport scientist who is performing the enrolling.

### Concealment mechanism [16b]

Allocation is concealed since two separate sport scientists are responsible for randomization and the participant enrolment, respectively. The information about the treatment allocation will be given to the sport scientist who is performing the diagnostics not until t1. Therefore, there is no influence on the assignment.

### Implementation [16c]

The generating of the allocation sequence, enrollment of the participants, and assignment of the participants to the interventions will be all done by separate sport scientists.

## Assignment of interventions: blinding

### Who will be blinded [17a]

Since the two interventions are completely different in their implementation, the intervention is impossible to blind. We are aware that there might be some bias due to this. Statistical analysis will be blinded, since it will be performed by a biostatistician.

### Procedure for unblinding if needed [17b]

Not applicable.

## Data collection and management

### Plans for assessment and collection of outcomes [18a]

#### Primary outcome

The *CAIT* is a questionnaire to get a subjective insight into regional functional impairments following LAS. It consists of 9 items measuring the severity of functional ankle instability [44]. The total score ranges from 0 to 30 with 0 representing a painful and strongly instable ankle during low-intense everyday activities and 30 representing a pain-free and subjectively stable ankle. Furthermore, the CAIT score is one out of three criteria, established by the International Ankle Consortium, to define CAI [8]. The Minimal Clinical Important Difference (MCID) of the CAIT is reported to be ≥ 3 points [43].

#### Secondary outcome

The *FAAM* is a questionnaire to assess the subjective impairments of ankle joint function [45]. It has 29 items divided into two subscales, a 21-item Activities of Living Subscale, and an 8-item Sport Subscale. Higher scores represent higher levels of function for each subscale, with 100% representing no dysfunction. The MCIDs are 8 and 9 points, respectively [46].

The questionnaires (CAIT and FAAM) are assessed by the web-service of Heartbeat medical (https://heartbeat-med.com/de/), which collects patient data via tablet, or any device at home (via email invitation). Biomechanical tests take place in the motorics laboratory “Athletikum Rhein Ruhr” affiliated to the department for arthroscopic surgery, sportstraumatology, and sportsmedicine, BG Klinikum Duisburg.

*Gait and run analysis* will be performed at 5 and 10 km/h, respectively. The analysis time interval will be 30 s each. Inertial measurement units (IMU) and EMG sensors are attached to measure three-dimensional kinematic data as well as muscle activity (see detailed descriptions below). The gait and run analyses take place on a motorized treadmill (h/p/cosmos sports & medical GmbH, Nussdorf-Traunstein, Germany) with an integrated pressure plate (FDM-THQ 2i, Zebris medical GmbH, Isny, Germany) sampling data at 300 Hz.

**Fig. 2:**
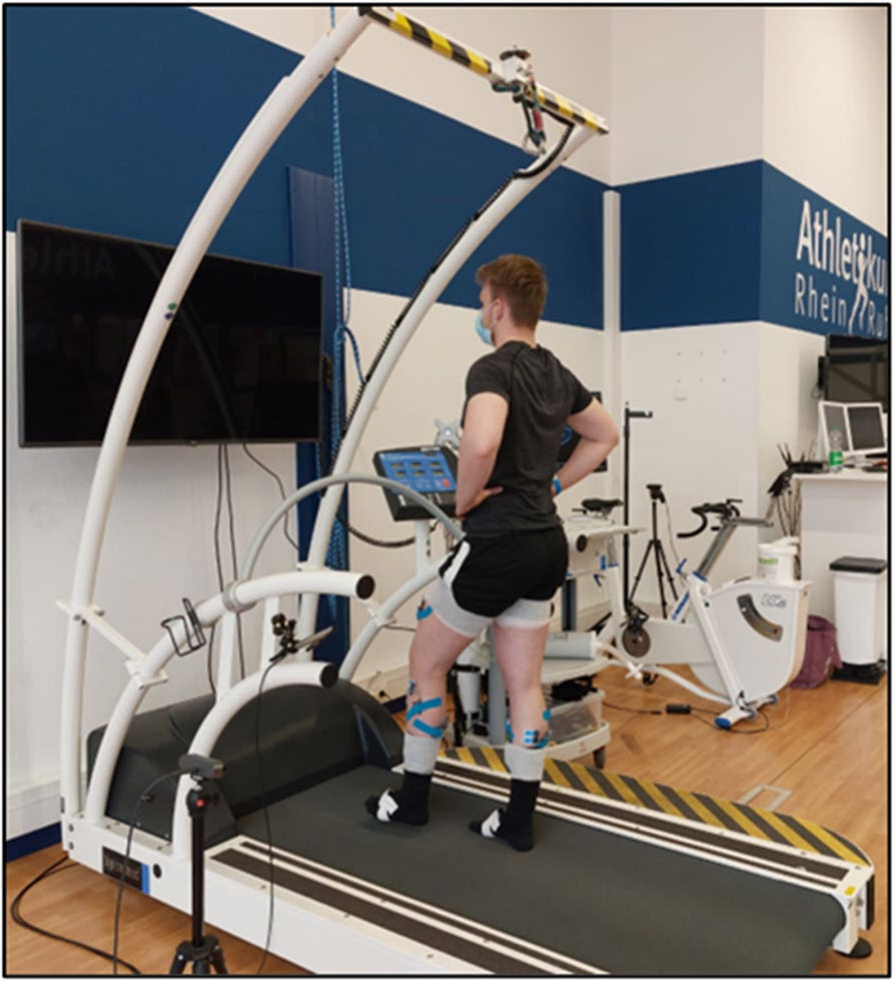
Single-leg stance

*Static postural control* is measured on the pressure plate of the treadmill. Trials of single-leg stance with eyes open and closed are performed with each leg (10 s each) (Fig. 2). For analysis, the COP is measured. We will track the COP area (mm^2^), mean COP velocity (mm/s), and the COP excursion length (mm). Additionally, there will be TTB analysis during the assessment of static balance. TTB measures estimate the time it would take for the COP to reach the boundary of the base support (individual foot boundaries) if the COP would continue to move on its trajectory at its instantaneous velocity. A lower TTB indicates greater postural instability as the COP is reaching its boundary of the base support faster [13]. Trials are repeated if the participants lose balance during the single-leg stance.

**Fig. 3:**
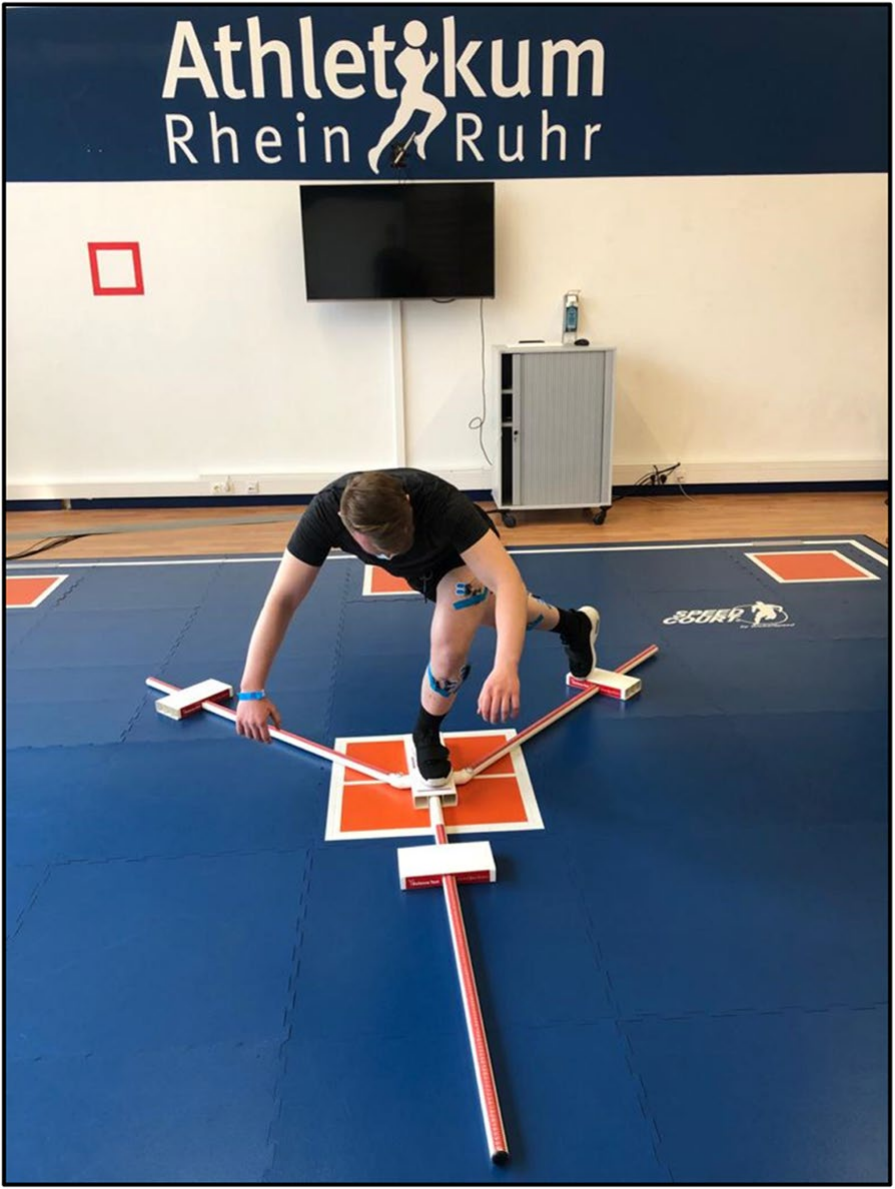
Modified Star Excursion Balance Test

*Dynamic postural control* is assessed via the mod. SEBT. Measurements (cm) are taken in the anterior, posterior-medial, and posterior-lateral reach direction. A standardized test kit is used. The kit consists of Y-shaped tubes connected by a central platform. Boxes placed on each tube allow the patient to slide into all three reaching directions allowing to assess reach distance by scales attached to the tubes (Fig. 3). The participants maintain a single-leg stance with their heels placed firmly on one of the three boxes of the test kit while sliding with the first of the other three boxes with the opposite leg as far as possible. Absolute differences between both legs as well as the composite score (summed average of right/left anterior, posterior-medial, and posterior-lateral values normalized to leg length) are determined.

**Fig. 4:**
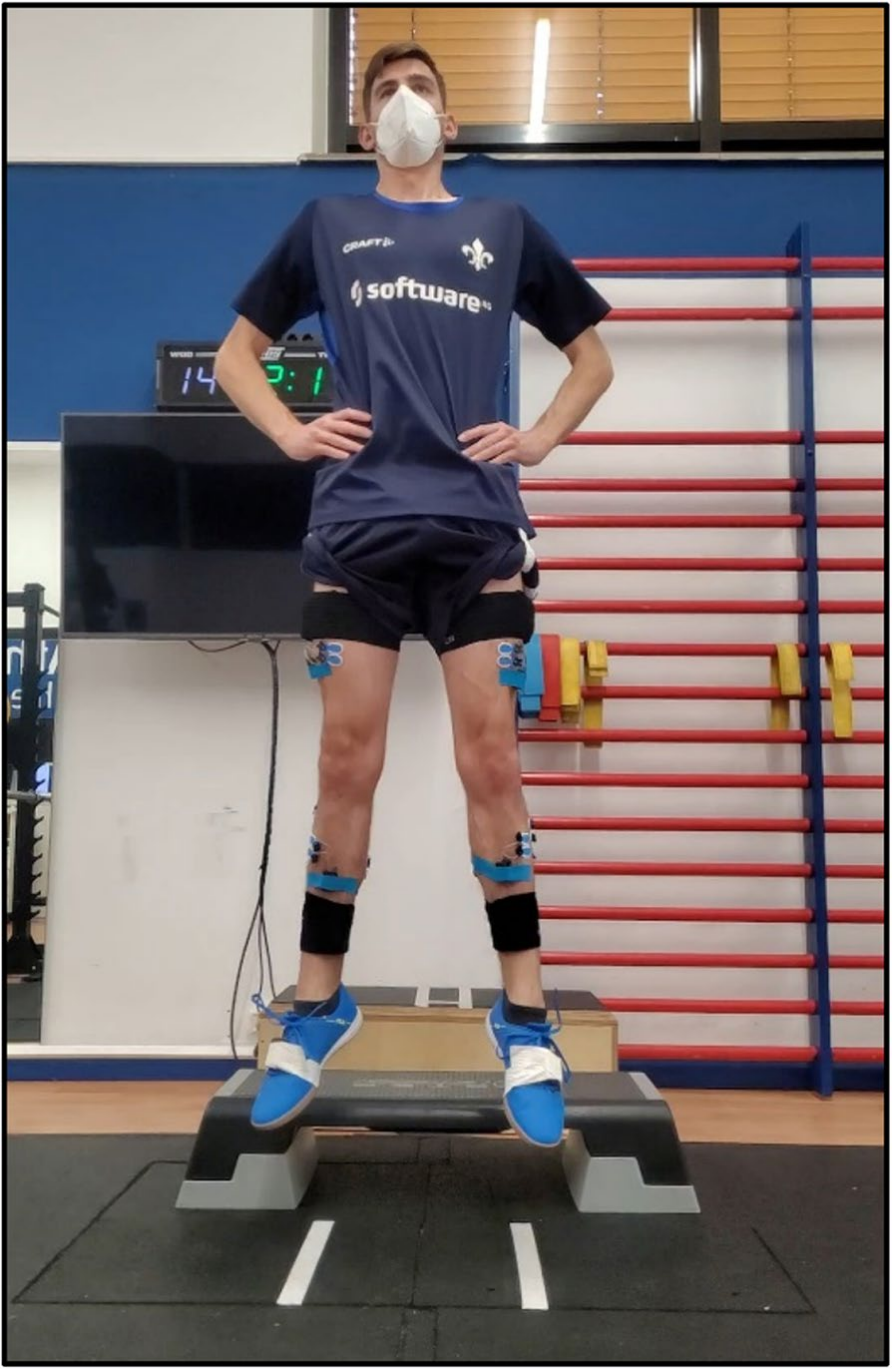
Dropjump

There are two types of *jumps* that are assessed: single-leg drop landing and bilateral drop-jump. Both jumps are performed from a 20 cm and 30 cm box onto two parallel independent force plates (FP-4060–08, Bertec Corporation, Columbus, Ohio, USA) to measure vertical ground reaction force. Data is sampled at 1500 Hz with a standard bandwidth filter of 500 Hz. The bilateral dropjump is a reliable test which is mainly determined by a person’s capability to utilize muscle–tendon elastic energy storage to generate force and can be used to assess maximum strength output, rate of force development and stiffness [47]. The participants are instructed to have a minimal ground contact time followed by a maximal rebound jump (Fig. 4).

**Fig. 5:**
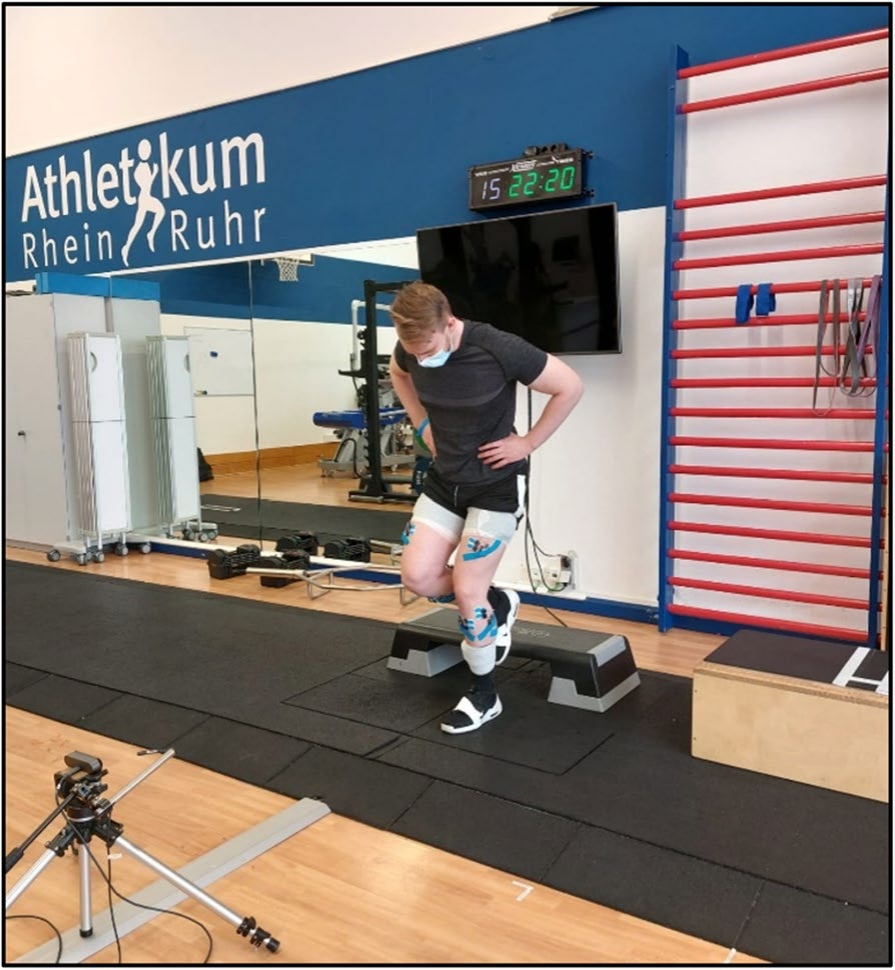
Single-leg drop landing

For the single-leg drop landing, the participants are instructed to drop forward onto the force plate, landing on one leg, resume, and hold a steady position for approximately 3 s. In both jumps, the participants are instructed to keep their hands fixed at their hips (Fig. 5). During the jump execution, it is important that the participants land within the boundaries of the force plates. If not, the jump will be repeated. There are going to be three single-leg drop landings for each side and per height as well as three drop-jumps per height. IMU and EMG sensors are attached to measure three-dimensional kinematic data as well as muscle activity (see detailed description below).

The *strength* diagnostic will be performed in a motorized dynamometer (IsoMed, D. & R. Ferstl GmbH, Hernau, Germany) which is able to measure bilateral force (N) production in a closed kinetic chain as well as plantarflexion and dorsiflexion torque (Nm) in an open kinetic chain. There will be isometric as well as isokinetic (concentric/eccentric) lower limb strength measurements and measurements of the plantar- and dorsiflexors (concentric/eccentric and eccentric/concentric). For all tests, the participants will be fixated with two shoulder restraints, one waist strap, and an additional restraint over the distal thigh during the plantarflexion/dorsiflexion to secure them firmly to the dynamometer and prevent compensatory movement.

**Fig. 6:**
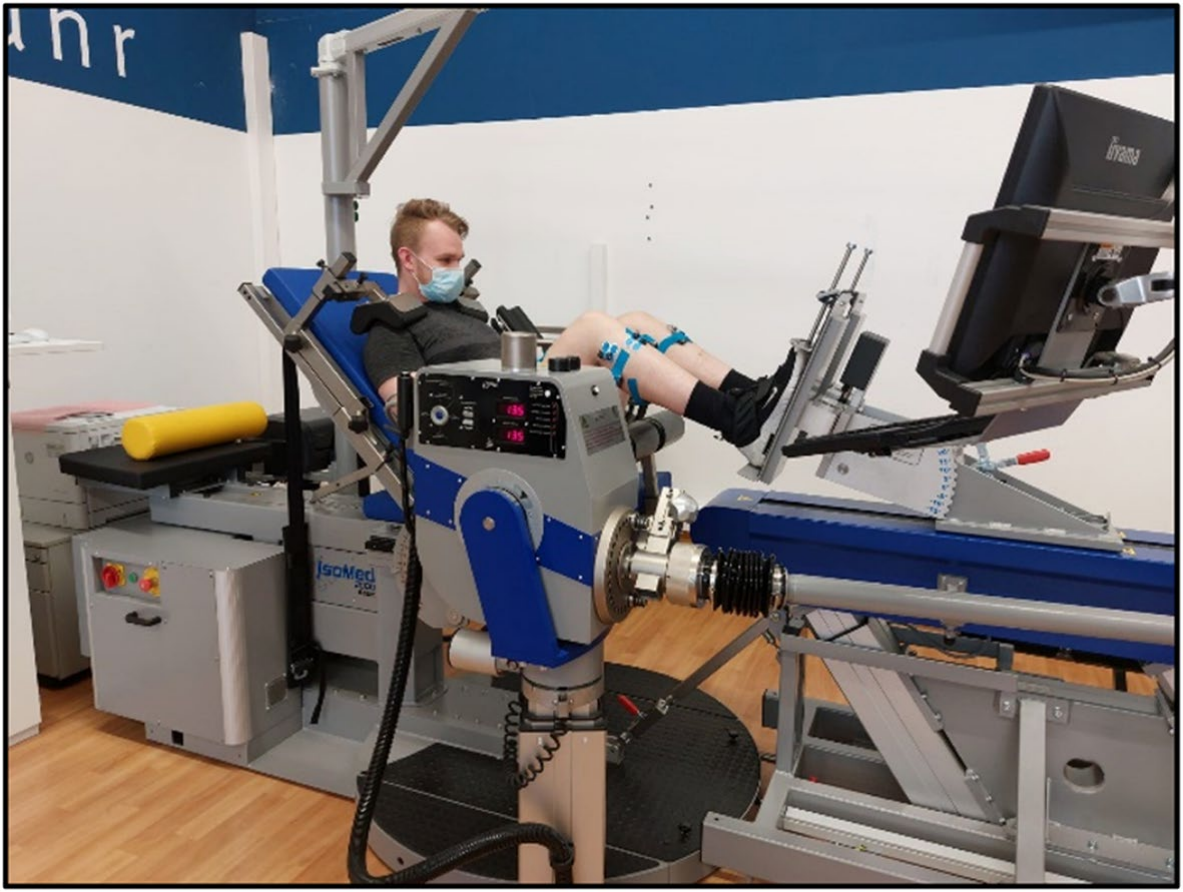
Legpress measurement

The legpress measurements include maximal isometric voluntary contractions (3 repetitions with 1 min rest) at 90° hip flexion, 90° knee flexion, and neutral foot position which is determined using a handheld goniometer as well as a dynamic (concentric extension/eccentric extension) test protocol (6 repetitions) with a range of motion (ROM) from 90° knee flexion to approximately 10° knee flexion (determined using handheld goniometer). The movement speed is set to 120 mm/s (Fig. 6).

**Fig. 7:**
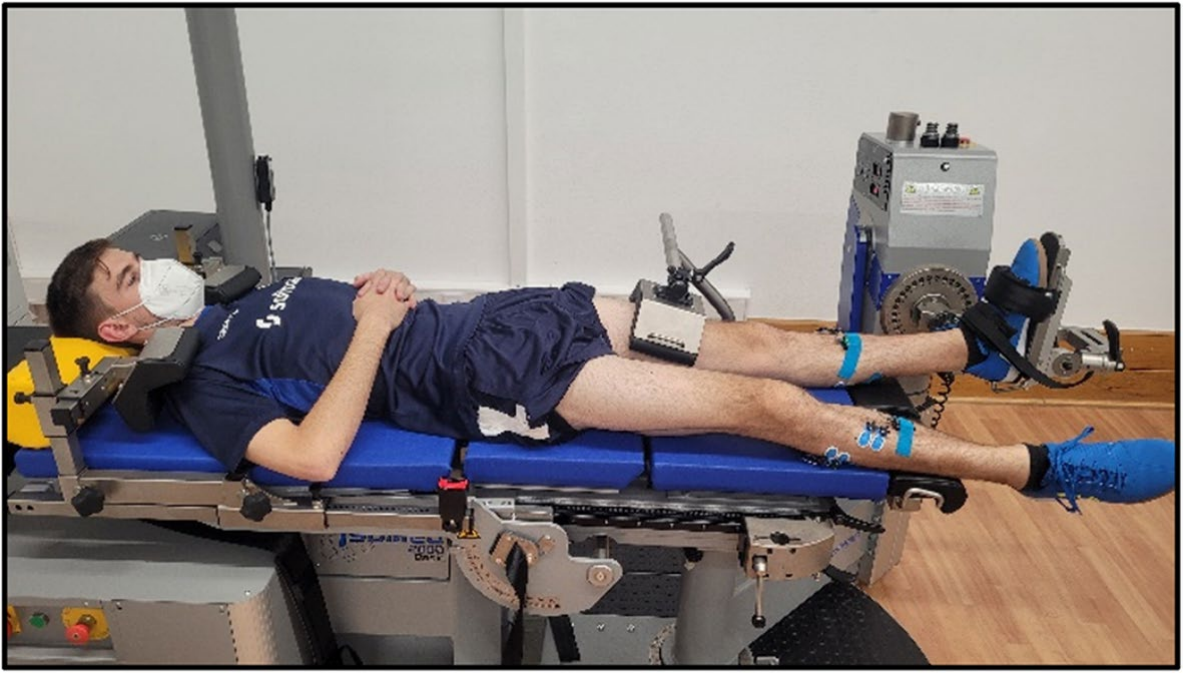
Isokinetic measurement

For the plantarflexion/dorsiflexion measurements, the participants lay in a supine position with their knee joints fully extended. The ankle joint’s axis of rotation is assumed to pass through the lateral malleolus and is visually aligned with the dynamometer’s axis of rotation. Axes alignment is performed at 90° ankle angle flexion (footplate relative to shank) which was determined using a handheld goniometer (Fig. 7). The test protocol comprises concentric plantarflexion and eccentric plantarflexion as well as eccentric dorsiflexion and concentric dorsiflexion with a velocity of 30°/s for both sides (4 repetitions each). The ROM is set to − 15° dorsiflexion to 20° plantarflexion.

The same setup is used to measure the active and passive *ROM* of the participants. Therefore, the ankle is moved into maximal plantarflexion (+ °) and dorsiflexion (− °) either voluntary by the participant (active ROM) or by the investigator (passive ROM). During the passive movement, the participant is instructed to keep his muscles as relaxed as possible.

*Joint repositioning sense* of the plantarflexion/dorsiflexion movement is tested according to the study of Konradsen and Magnusson as well as Sefton et al. [48, 49]. It is determined by the participant’s ability to actively replicate a passively placed joint reference angle. The ankle will be passively moved into neutral ankle angle (90°), determined with a handheld goniometer, where it remains for a few seconds. It then will be passively moved through two full range of motion cycles. The participant is asked to actively reproduce the neutral reference angle. Three practice trials are followed by six test trials. Constant error will be calculated as the actual difference between the reference angle and the matching angle, with + or − indicating the direction of the error. The variable error will be calculated as the standard deviation of the constant error, indicating the error occurring when matching the reference angle. The absolute error will be calculated as the absolute value of the difference between the matching and reference angle, indicating the composite of both the systematic and random error [48].

**Fig. 8:**
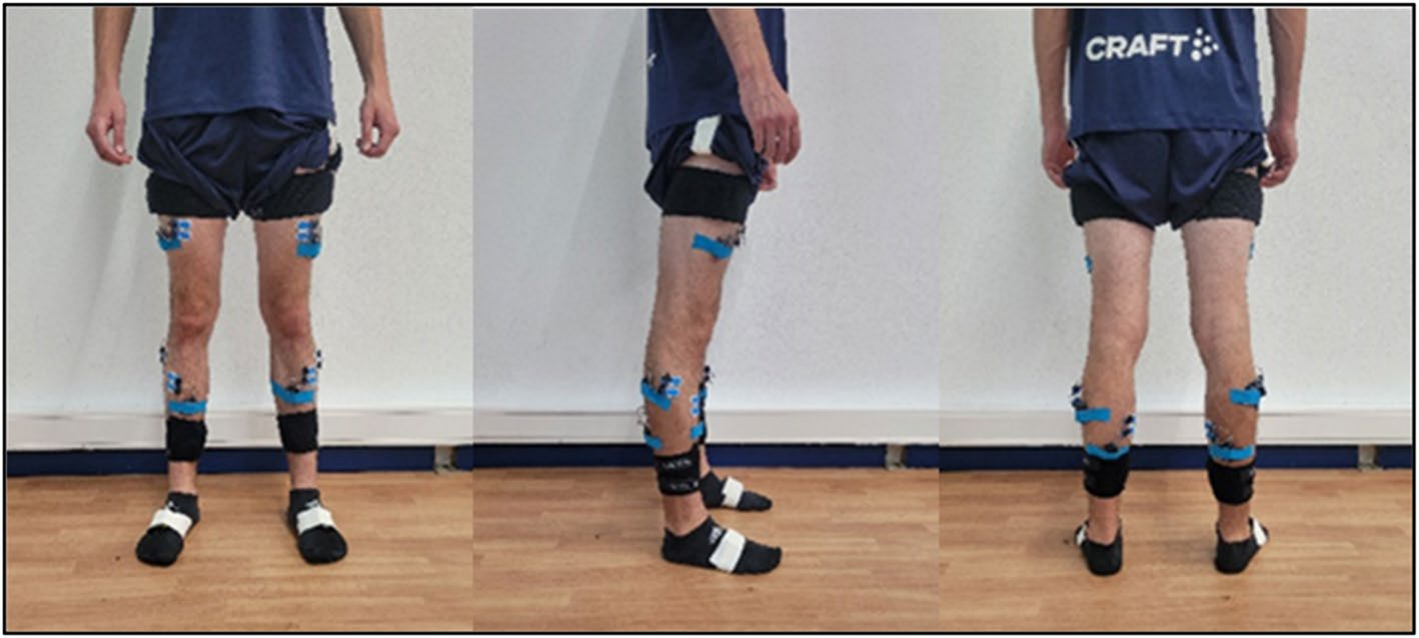
EMG and IMU placement

*Kinematic data* is collected at 200 Hz using a portable motion capture system (myoMotion, Noraxon U.S.A. Inc., Scottsdale, Arizona, USA). Therefore, data of IMU are telemetrically transferred to the measurement software (MR3.16.58, Noraxon U.S.A. Inc., Scottsdale, Arizona, USA). The software is able to detect steps as well as jumps based on the input systems. The set of IMUs contain a total of seven sensors which are placed on pelvis, both femurs, tibiae, and feet. Straps are used to fix the sensors tightly in these locations (Fig. 8). The sensors are further attached with cohesive tape to reduce soft tissue artifacts and sensor displacement (Elastomull, BSN Medical, Hamburg, Germany). The pelvic sensor is applied over the first sacral spinous process. Upper leg sensors are placed at about 70% length of the femur over the tensor fasciae latae. Lower leg sensors are fixed over the bony surface at about 50% length of the tibia. Feet sensors are placed over the upper foot, slightly below the ankle. Sensor calibration takes place in a neutral upright position and re-calibration will be performed before each task to minimize sensor drift and electromagnetic interference which reduces data quality.

*Electromyography* is performed using surface EMG. We use eight telemetric EMG sensors (TeleMyo DTS EMG, Noraxon U.S.A. Inc., Scottsdale, Arizona, USA) sampling data at 1500 Hz with a 10–500 Hz bandpass filter. EMG signals were further rectified and processed using a moving average root mean square (RMS) algorithm. Peroneus longus, tibialis anterior, gastrocnemius medialis, and rectus femoris are selected to analyze (Fig. 8). Two ECG surface electrodes (Ambu Blue Sensor N, Ambu GmbH, Bad Nauheim, Germany) per muscle are applied according to SENIAM guidelines. They are placed in parallel following the muscle fiber direction with an approximate inter-electrode difference of 2 cm. Skin preparation includes shaving of the defined area, followed by the removal of dead skin cells with an abrasive paste (Everi, Spes Medica, Battipaglia, Italy) and by cleaning with isopropyl alcohol. The EMG sensors are wired to the electrodes and attached to the skin with kinesiologic tape (PINO Pharmazeutische Präparate GmbH, Hamburg, Germany).

Raw data of IMU, EMG, treadmill, force plates, and the motorized dynamometer are synchronized in the measurement software.

### Plans to promote participant retention and complete follow-up [18b]

As already mentioned in 11b, there are multiple strategies to promote the participant’s adherence to the intervention. There will also be check-ups during the 6-week-intervention. These are video calls which are attractive to the participant since they provide a low-threshold access to the intervention. These check-ups help to guide the intervention and eventually adapt it. The 6-month follow-up will be done via email with Heartbeat (service to collect questionnaires). By this, the participants can answer the questionnaires from home. Heartbeat will send reminders to the participant in case of not answering the questionnaire. There will be a list of all expected outcomes for the patients. The participants will get an expense allowance after 6 weeks and 12 months. By dividing disbursement of the expense allowance to the 6-week and 12-month time points, patients are further encouraged to complete their follow-up. In case a participant does not want to complete the follow-up, especially the diagnostic, we give the option to just complete the questionnaires via Heartbeat.

### Data management [19]

Experimental data of the measurement software (gait/run analysis, jump analysis, static postural control, and strength measurement) will be exported individually into excel files right after data collection (Microsoft Excel 2016, Redmond, USA). SEBT data, ROM, strength measurements of the peroneus muscle, and measurements of the joint positioning sense testing will be documented by paper forms, created by the study team, which will later be transferred to electronic. Hard-copy data of the paper-forms will be stored for a period of 10 years after completion of the study in separate file folders. Proms (CAIT & FAAM) are collected via Heartbeat medical APP (service to collect questionnaires) which can also be transferred into excel files after checking the data. All data will be transferred into a prepared mother table. Everything is stored on two secure servers within the hospital computer system. Only the study team will be given access to the study folders where the database is saved. Further, a password system will be utilized to control access. All the data are checked before being transferred to the statistical software for analysis. Data integrity will be enforced by referential data and range checks by two separate sport scientists. Within the ethics approval, there is an individual data protection policy on disclosure/ data collection, data management, data labelling, and data protection.

### Confidentiality [27]

Data will be kept pseudonymized with an individual trial identification number in folders with limited access, only by members of the trial team to ensure confidentiality. All records that contain names or other personal identifiers will be stored separately from study records. All local databases will be secured with password-protected access systems.

### Plans for collection, laboratory evaluation, and storage of biological specimens for genetic or molecular analysis in this trial/future use [33]

Not applicable as there is no laboratory evaluation or biological specimen collection in this study.

## Statistical methods

### Statistical methods for primary and secondary outcomes [20a]

As introduced earlier, there is a primary aim and a secondary aim of the study. While the primary aim is investigated as a longitudinal study, the secondary aim of the study is to have a comparison from injured to non-injured participants. Therefore, statistical analysis of both approaches differs:

#### Primary study aim

The primary outcome, CAIT, will be analyzed with a repeated-measurement ANOVA using JASP (JASP 2018, Amsterdam, Netherlands). The same will be done for some secondary outcome measurements like FAAM, postural control, joint repositioning sense, jump, and strength metrics.

Continuous data (isokinetic measurements, IMU and EMG data of gait, run and jumps, ground reaction forces) will be analyzed via SPM. Most statistical test procedures are often based on the assumption of simple Gaussian distribution and reduce data complexity by comparing maxima, minima, or mean data. Instead of reducing data complexity and the underlying informational content of continuous variables, SPM allows for probalistic inferences regarding any kind of smooth, bounded continuous parameters [50, 51]. All SPM analyses are implemented using the open source spm1d code (v.M0.1, www.spm1d.org) in Matlab (R2014a, 8.3.0.532, The Mathworks Inc, Natick, MA).

#### Secondary study aim

The primary study outcome, CAIT, will be analyzed with an unpaired *t*-test, comparing injured patients with the uninjured control group. The same will be done for some secondary outcome measurements like FAAM, postural control, joint repositioning sense, jump, and strength metrics.

Continuous data (isokinetic measurements, IMU and EMG data of gait, run and jumps, ground reaction forces) will be analyzed via SPM, as described above.

### Interim analyses [21b]

Not applicable.

### Methods for additional analyses (e.g., subgroup analyses) [20b]

Participant data will be split into age groups for additional analysis. Previous work of a retrospective analysis has been shown that there was the highest CAI rate in the oldest age group (14–25 years vs 26–40 years vs 41–55 years) [26]. Therefore, it might be of interest to see if this distribution changes by the training intervention and how age groups differ in functional parameters. Another possibility to divide subgroups is different levels of activity. It could be higher levels of activity might serve as a protection factor for developing a CAI.

### Methods in analysis to handle protocol non-adherence and any statistical methods to handle missing data [20c]

Again, statistical methods to handle non-adherence and missing data must be separated for the primary and secondary study aim:

#### Primary study aim

For the primary study aim, participants who withdraw from the study will excluded from statistical analysis.

#### Secondary study aim

Since the secondary study aim is a cross-sectional analysis of t1 and t2, later withdrawal is irrelevant for the analysis. Therefore, analysis will be conducted on an intent-to-treat basis, including data for each participant who was randomized, regardless of whether they later withdraw from the study.

### Plans to give access to the full protocol, participant-level data, and statistical code [31c]

Data will not be released to any third party and will be analyzed independently by the study team. Data will be made available upon request to the primary investigator.

## Oversight and monitoring

### Composition of the coordinating center and trial steering committee [5d]

The study team (consisting of three sport scientists, a research manager, and a physician) is responsible for the day-to-day conduct of the trial and will meet regularly at least quarterly, across the course of the trial, making all relevant decisions regarding the study. The sport scientists specifically are in charge of patient enrolment and conduction of the 6-week SMART-intervention as well as the diagnostics. The physician is responsible for the clinical assessment of the patients during patient enrolment. The study team itself is in charge of the project administration.

### Composition of the data monitoring committee, its role and reporting structure [21a]

The trial will be monitored by the study investigators. There will be regular check-ups with the participants during the intervention to eventually adapt the training protocol.

A formal data monitoring committee is not needed for the current trial. This is because the participants are not exposed to significant harms and because of its short duration (6 weeks).

### Adverse event reporting and harms [22]

The investigators stay in regular contact with the participants. Adverse events, like recurrent ankle sprains, will be documented at each follow-up visit. Recurrent ankle sprains have an influence on the chronic instability of the ankle and will therefore be reported in future publications. Participants are asked to report significant worsening of their ankle by contacting the study team immediately, so that physical examinations can be guaranteed.

### Frequency and plans for auditing trial conduct [23]

There are no plans for independent auditing of trial conduct.

### Plans for communicating important protocol amendments to relevant parties (e.g., trial participants, ethical committees) [25]

Any change to the trial protocol will be communicated with the ethical committees and trial registry.

### Dissemination plans [31a]

We will disseminate our research with academic and professional audiences via presentations at conferences and publication of findings in high-impact journals.

## Discussion

The study has two main aims which are going to address: The primary aims are to investigate the effectiveness of a sensorimotor training intervention in contrast to standard therapy after an acute LAS on perceived ankle joint function. We hypothesize that patients treated with SMART show a better subjective outcome in perceived ankle joint function than patients treated with NORMT. Since currently, there is a high rate of acute LAS developing a CAI, this study has the potential to improve the healthcare for LAS patients in the long term. The data of this study might therefore be used for a future standardized evidence-based rehabilitation-concept being able to reduce the long-term CAI rate.

The secondary aim is to determine an effective test battery for the valid measurement of a functional ankle instability. This will be achieved by comparing the injured patients with an uninjured control group. Therefore, it might be necessary to not only compare the acute state (t1) with the uninjured control group but also the time point after the intervention (t2). The goal is to identify specific tests which are able to display an abnormal trend of the ankle function. These would then further allow to navigate through LAS rehabilitation of future patients.

Additionally, it might be possible to identify risk factors in the test battery predicting a CAI. Eventually, some patients have a higher probability to develop a CAI. These identified risk factors could then be used to screen patients after the injury and monitor them more closely.

## Trial status

The recruitment started on 3 January 2022, and the trial protocol is still used in its original version. The recruitment phase is estimated to be completed in January 2024.

## Supplementary Information

**Additional file 1: Supplementary Figure 1. **Training program – Exercise overview. **Supplementary Figure 2.** Training program – Modification of duration and intensity. **Supplementary Figure 3. **Training program - Modification of exercise difficulty.

## Data Availability

The data is maintained on a hospital server that only the investigators will have access to.

## Abbreviations

CAIT: Cumberland Ankle Instability Tool
COP: Center of pressure
FAAM: Foot and Ankle Ability Measure
LAS: Lateral ankle sprain
MVC: Maximal voluntary contraction
ROM: Range of motion
SEBT: Star Excursion Balance Test
SPM: Statistical parametric mapping
TTB: Time-to-boundary
