# Supplementary material for: Effectiveness of the SMART training intervention on ankle joint function in patients with first-time acute lateral ankle sprain: study protocol for a randomized controlled trial
Source: Trials. 2023 Mar 3;24:162. doi: 10.1186/s13063-023-07195-2 (PMC9985260; doi:10.1186/s13063-023-07195-2)
Supplement: Supplementary file 1 — Additional file 1: Supplementary Figure 1. Training program – Exercise overview. Supplementary Figure 2. Training program – Modification of duration and intensity. Supplementary Figure 3. Training program - Modification of exercise difficulty. [file 13063_2023_7195_MOESM1_ESM.docx]

**Supplementary material**

| Week | Exercise domain | | | | |
| --- | --- | --- | --- | --- | --- |
|  | S | M | A | R | T |
| 1 | 1,2 | 1,2 | 1,2,3,4,5,6,7,8,9 | 1,2,3,4 | 1,2 |
| 2 | 1,2 | 1,2 | 1,2,3,4,5,6,7,8,9 | 1,2,3,4 | 1,2 |
| 3 | 1,2 | 1,2 | 1,2,7,9,10,11 | 1,2,4,5,6,7 | 3,5 |
| 4 | 1,2 | 1,2 | 1,2,7,9,10,11 | 1,2,4,5,6,7 | 3,5 |
| 5 | 1,2 | 1,2 | 1,2,7,10,11 | 1,2,4,5,6,7 | 4,5,6 |
| 6 | 1,2 | 1,2 | 1,2,7,10,11 | 1,2,4,5,6,7 | 4,5,6 |

**Supplementary Figure 1.** Training program – Exercise overview

| Week | Duration | | | | |
| --- | --- | --- | --- | --- | --- |
|  | S | M | A | R | T |
| 1 | 2 x 20 sec | 2 x 20 sec | 2 x 20 sec | 2 x 40 sec | 1 x 40 sec |
| 2 | 2 x 20 sec | 2 x 20 sec | 3 x 20 sec | 2 x 50 sec | 1 x 60 sec |
| 3 | 2 x 20 sec | 2 x 20 sec | 2 x 30 sec | 2 x 40 sec | 3 x 20 sec |
| 4 | 2 x 20 sec | 2 x 20 sec | 3 x 30 sec | 2 x 40 sec | 4 x 20 sec |
| 5 | 2 x 20 sec | 2 x 20 sec | 2 x 40 sec | 3 x 40 sec | 5 x 20 sec |
| 6 | 2 x 20 sec | 2 x 20 sec | 3 x 40 sec | 3 x 40 sec | 6 x 20 sec |

**Supplementary Figure 2.** Training program – Modification of duration and intensity

|  | Level 1 | Level 2 | Level 3 |
| --- | --- | --- | --- |
| S1 | Toe-pulls (linear) | Bend support leg | Eyes closed |
| S2 | Toe-pulls (diagonal) | Bend support leg | Eyes closed |
| M1 | Ankle Rotations | Bend support leg | Eyes closed |
| M2 | Knee Rotations | Bend support leg | Eyes closed |
| A1 | Walking on Airex | Skippings on Airex | Knee Lift on Airex |
| A2 | Squat on Airex | Squat variation on Airex | Squat variation on Airex |
| A3 | Towel Curl | − | − |
| A4 | Towel Pick-up | − | − |
| A5 | Single Leg Stance on Airex with Pull-down | − | − |
| A6 | Single Leg Stance on Airex with Butterfly-Reverse | − | − |
| A7 | Single Leg Stance on Airex with eyes closed | Head motions | Bend support leg and head motions |
| A8 | Single Leg Stance on Airex: foot tap | − | − |
| A9 | Single Leg Stance: Hip Hinge, Arm Reach, Knee Lift | − | − |
| A10 | Single Leg Stance on Airex: rapid arm movements in transverse plane (like opening a door) | Eyes closed | − |
| A11 | Single Leg Stance: Bottle around body | Single Leg Stance: bounce ball | Single Leg Stance: bounce ball against wall |
| R1 | Resisted Plantarflexion | Heel Raises | Toe Walks |
| R2 | Resisted Dorsiflexion | Toe Raised Walking | − |
| R3 | Resisted Eversion | − | − |
| R4 | Hip Abduction | Monster Walk | − |
| R5 | Hindu Squats | Hindu Squats with load | − |
| R6 | Deadlifts | Single Leg Deadlifts | − |
| R7 | Bulgarian Split Squats | Single Leg Squats | − |
| T1 | Linear Stepups on Airex | Linear Hurdles | − |
| T2 | Lateral Stepups on Airex | Lateral Hurdles | − |
| T3 | Linear Jumps | − | − |
| T4 | Lateral Reactive Jumps | − | − |
| T5 | Lateral Jumps | Increased velocity | − |
| T6 | Linear Reactive Jumps | − | − |

**Supplementary Figure 3.** Training program - Modification of exercise difficulty
